# Risk factors associated with the recurrence of diabetic foot ulcers: A meta-analysis

**DOI:** 10.1371/journal.pone.0318216

**Published:** 2025-02-14

**Authors:** Chunmei Lin, Jianqing Tian, Zhijun Zhang, Caiyu Zheng, Jinhao Liu

**Affiliations:** 1 Department of Endocrinology and Metabolism, Fujian Medical University Xiamen Humanity Hospital, Xiamen, Fujian Province, People’s Republic of China; 2 Department of Vascular Surgery, Zhongshan Hospital Affiliated to Xiamen University, Xiamen, Fujian Province, People’s Republic of China; Universitas Muhammadiyah Aceh, INDONESIA

## Abstract

**Aims:**

Diabetic foot ulcers have caused significant medical, economic and social consequences for patients, families and society. With appropriate treatment, many diabetic foot ulcers can heal, temporarily avoiding possible amputation. Unfortunately, even if foot ulcers subside, recurrence is still common. The recurrence of ulcer has brought another physical and psychological trauma to diabetic foot patients who yearn for a better life. Therefore, it may be more useful to evaluate the factors associated with ulcer recurrence in diabetic foot ulcer patients.

**Methods:**

The PubMed, Web of Science, Embase and China National Knowledge Infrastructure databases were comprehensively searched for prospective or retrospective studies published up to February 1, 2024. All English or Chinese language studies on diabetic foot ulcer patients who experience recurrence were included, and RevMan 5.3 software was used to analyze the data.

**Results:**

A total of 22 studies meeting the eligibility criteria were ultimately included in this meta-analysis. 1861 of 5252 diabetic foot ulcer patients experienced recurrence during follow-up. The following variables were associated with an increased risk of ulcer recurrence: male (OR = 1.26, 95% CI = 1.10 ~ 1.44, P = 0.0009), smoking history (OR = 1.18, 95% CI = 1.04 ~ 1.35, P = 0.01), living alone (OR = 1.86, 95% CI = 1.21 ~ 2.86, P = 0.004), plantar ulcers (OR = 2.44, 95% CI = 1.41 ~ 4.23, P = 0.001), diabetic retinopathy (OR = 1.59, 95% CI = 1.35 ~ 1.88, P < 0.00001), diabetic nephropathy (OR = 1.37, 95% CI = 1.12 ~ 1.68, P = 0.002), diabetic peripheral neuropathy (OR = 1.78, 95% CI = 1.45 ~ 2.19, P < 0.00001), foot deformity (OR = 2.51, 95% CI = 1.85 ~ 3.40 P < 0.00001) and peripheral arterial disease (OR = 3.10, 95% CI = 2.43 ~ 3.95 P < 0.00001). However, hypertension (OR = 1.16, 95% CI = 0.96 ~ 1.40, P = 0.13) and body mass index (MD = 0.20, 95% CI = −0.12 ~ 0.53, P = 0.22) were not associated with diabetic foot ulcer recurrence.

**Conclusions:**

Our meta-analysis identified the following important risk factors for diabetic foot ulcer recurrence: male sex, smoking history, living alone, plantar ulcer, diabetic retinopathy, diabetic nephropathy, diabetic peripheral neuropathy, foot deformity, and peripheral arterial disease. Understanding these factors and their impact on ulcer recurrence is crucial for multidisciplinary teams to develop management and treatment plans for diabetic foot ulcer patients.

## Introduction

Diabetes is one of the most common endocrine diseases, and its complications lead to considerable challenges for the life and survival of patients. More than 537 million people aged between 20 and 79 years worldwide have diabetes, and the number of cases is still rising [1,2]. Diabetic foot ulcer (DFU) is the main reason for hospitalization among diabetes patients and one of the most common, serious and expensive complications of diabetes. DFUs cause significant medical, economic and social consequences for patients, their families and society [3]. It is estimated that DFUs affect about 18.6 million people worldwide, and about 1.6 million people in the United States every year [4]. Among these ulcer patients, approximately 20% will undergo a lower limb amputation, either minor (i.e., part of the foot) or major (i.e., above foot) [5].

With proper treatment, many DFUs can heal, thus avoiding possible amputations. Although about 30% to 40% of diabetes foot ulcers heal within 12 weeks. The recurrence rate is estimated to be 42% in one year and 65% in five years after healing [3,6]. Therefore, it may be more useful to think that patients with closed wounds are in remission rather than cured. A history of foot ulcers is considered to be one of the strongest predictors of future foot ulcers in diabetes [7–9]. Because of the high risk of infection, hospitalization and amputation, prevention of recurrence is one of the most important topics in the current treatment of DFU. If preulcer lesions are found in a time manner, treatment may prevent the recurrence of many ulcers. To address these issues, we conducted a meta-analysis to evaluate factors associated with ulcer recurrence in patients with DFU.

## Methods

This meta-analysis was conducted in accordance with the Preferred Reporting Items for Systematic Reviews and Meta-analyses (PRISMA) statement.

### Search strategy

The PubMed, Web of Science, Embase and China National Knowledge Infrastructure databases were searched up to February 1, 2024. The following keywords were used to search for studies examining DFUs: “Diabetic Feet” OR “Diabetic Foot” OR “Foot Ulcer, Diabetic”. The following keywords were used to search for studies examining risk factors: “Predictive factors” OR “Predictive factor” OR “Risk Factors” OR “Risk Factor” OR “Population at Risk” OR “Predictors”. The following keywords were used to search for studies examining recurrence: “recurrence” OR “recurrences” OR “recurrent” OR “recurrently” OR “relapse” OR “relapses” OR “recrudescence”. The three authors (Liu J, Lin C and Tian J) independently screened the studies. Disagreements among the reviewers were resolved through discussion.

### Inclusion and exclusion criteria

The inclusion criteria were as follows: (i) articles were prospective or retrospective studies based on original data; (ii) articles were published in the English or Chinese language; (iii) all patients were diagnosed with DFU, regardless of diabetes type; (iv) all patients were diagnosed with DFU, with or without history of amputation or ulcer history; and (v) data regarding the demographics and clinical characteristics of the DFU patients were available. The exclusion criteria were as follows: (i) reviews, letters to the editor, commentaries and editorials, irretrievable articles, animal studies and other studies from which patient data could not be extracted; (ii) studies for which the full text was not written in English or Chinese; and (iii) simple diabetic foot patients without ulcers or simple diabetic foot infection patients. Two independent authors (Zhang Z and Lin C) screened all titles and abstracts to determine the eligibility of the studies. Full texts were reviewed when eligibility could not be determined based on the abstracts, and any disagreements between the reviewers were resolved through discussion. All studies identified in the literature search were imported into Endnote X20 software.

### Data extraction and quality assessment

Two authors (Liu J and Lin C) independently extracted the following data from the included articles into structured tables: first author, year of publication, country and region, research design, number of cases, incidence, potential risk factors and corresponding data. Two researchers (Zheng C and Zhang Z) independently evaluated the quality of studies using the Newcastle–Ottawa Scale (NOS). The NOS was used to assess the risk of bias across three major domains: (i) “group selection”, up to 4 points; (ii) “comparability”, up to 2 points; and (iii) “assessment of outcome or exposure”, up to 3 points. The total NOS score of each study ranged from 0 to 9 [10], and studies were considered high-quality if they had scores of at least 5.

### Statistical analysis

Statistical analysis was performed using RevMan 5.3 software. The results are presented as the mean difference (MD) or odds ratios (ORs) with 95% confidence intervals (CIs), and a P value < 0.05 was considered to indicate statistical significance unless otherwise specified. In addition, heterogeneity was quantified using the Q test and I^2^ statistics. When the heterogeneity test indicated no significant heterogeneity (P > 0.1 and I^2^ < 50%), a fixed effects model was applied; otherwise, a random effects model was used. Begg’s funnel plot test was used to assess possible publication bias.

## Results

**Fig 1:**
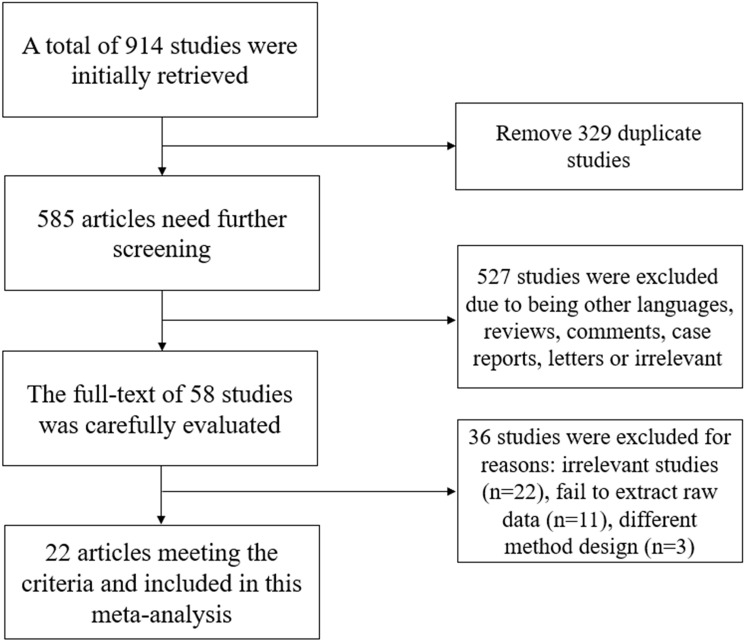
The selection process flow chart for the meta-analysis.

After systematically searching the databases, 914 studies were initially retrieved. After removing duplicate studies, 585 articles remained for screening. A total of 110 studies were excluded due to not including relevant data or being published in a language other than English or Chinese. Then, the titles and abstracts of the remaining studies were carefully screened, and 417 studies were excluded due to being comments, case reports, letters or irrelevant. After carefully reading and analyzing the full texts of the remaining 58 articles, a total of 22 studies meeting the eligibility criteria were ultimately included in this meta-analysis [11–32]. This meta-analysis included a total of 5252 patients with DFU, and 1861 patients experienced DFU recurrence during follow-up. Table 1 summarizes the basic characteristics of the included studies. The flow chart of the selection of studies included in the meta-analysis is shown in Fig 1.

**Table 1:** Basic characteristics of the included studies.

Author	Year	Study design	Country/Region	Sample size (RDFU/total)	Rate %	Quality assessment
L. Giurato [11]	2023	retrospective	Italy	47/127	37.01	6
H. Tan Dat [12]	2023	retrospective	Vietnam	17/57	29.82	6
Zhang, L. [13]	2023	prospective	China	31/120	25.83	6
Gong, H. [14]	2022	retrospective	China	315/817	38.56	6
Lv, J. [15]	2022	prospective	China	12/62	19.35	7
Gazzaruso, C. [16]	2021	retrospective	Italy	172/464	37.07	6
Cheng, Y. [17]	2021	retrospective	China	178/573	26.45	6
Tabanjeh, S.F. [18]	2020	retrospective	Jordanian	76/130	58.46	5
Shen, J. [19]	2020	retrospective	China	87/185	47.03	6
Hicks, C.W. [20]	2020	prospective	USA	117/304	38.49	7
Freitas, F. [21]	2020	prospective	Brazil	23/35	65.71	7
Cheng, Y. [22]	2019	retrospective	China	112/520	21.54	6
An, J. [23]	2019	retrospective	China	41/210	19.52	6
Khalifa, W.A. [24]	2018	prospective	Egypt	57/93	61.29	7
Mo, Z. [25]	2018	retrospective	China	338/789	42.84	6
Xie, Z. [26]	2018	retrospective	China	16/74	21.62	6
Waaijman, R. [27]	2014	prospective	Netherlands	71/171	41.52	7
Hu, X. [28]	2014	retrospective	China	213/574	37.11	6
Dubsky, M. [29]	2013	prospective	Czech Republic	42/73	57.53	7
Gonzalez, J.S. [30]	2010	retrospective	USA	95/333	28.53	8
Peters, E.J. [31]	2007	prospective	USA	49/81	60.49	8
Jude, E.B. [32]	2001	retrospective	UK	23/48	47.92	5

Abbreviation: RDFU, Recurrent diabetic foot ulcer.

### Sex

**Fig 2:**
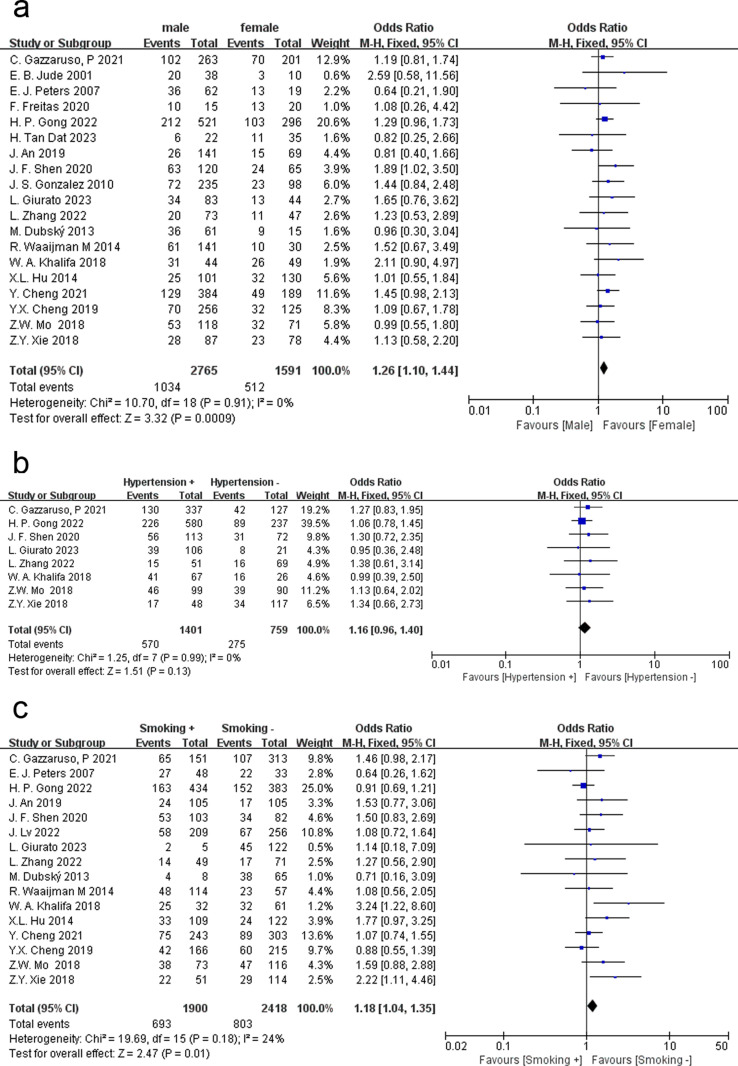
Meta-analysis results for the recurrence of DFUs between the two groups. (a) Sex; (b) Hypertension; (c) Smoking history.

Sex was analyzed using a fixed effects model (P = 0.91, I^2^ = 0%). The recurrence rate of foot ulcers in male DFU patients was 37.39%, and that in female DFU patients was 32.18%. The analysis results show that male patients with DFU had a significantly higher incidence of recurrence (OR = 1.26, 95% CI = 1.10 ~ 1.44, P = 0.0009) (Fig 2a).

### Hypertension

In this study, hypertension was defined as systolic blood pressure ≥ 140 mmHg, diastolic blood pressure ≥ 90 mmHg, or the use of antihypertensive medication. A fixed effects model was used for data analysis (P = 0.99, I² = 0%). Eight related studies were included in the analysis, and the results showed that recurrence after DFU healing was not related to hypertension (OR = 1.16, 95% CI = 0.96 ~ 1.40, P = 0.13) (Fig 2b).

### Smoking history

Smoking was common in patients with DFU. Therefore, we evaluated the impact of smoking history on ulcer recurrence in DFU patients. Smoking history was defined as smoking in the past or present. Due to the low heterogeneity of the data (P = 0.18, I² = 24%), a fixed effects model was used for analysis. Across 16 articles, we found that smoking history may increase the risk of recurrence in DFU patients (OR = 1.18, 95% CI = 1.04 ~ 1.35, P = 0.01) (Fig 2c).

### Living alone

**Fig 3:**
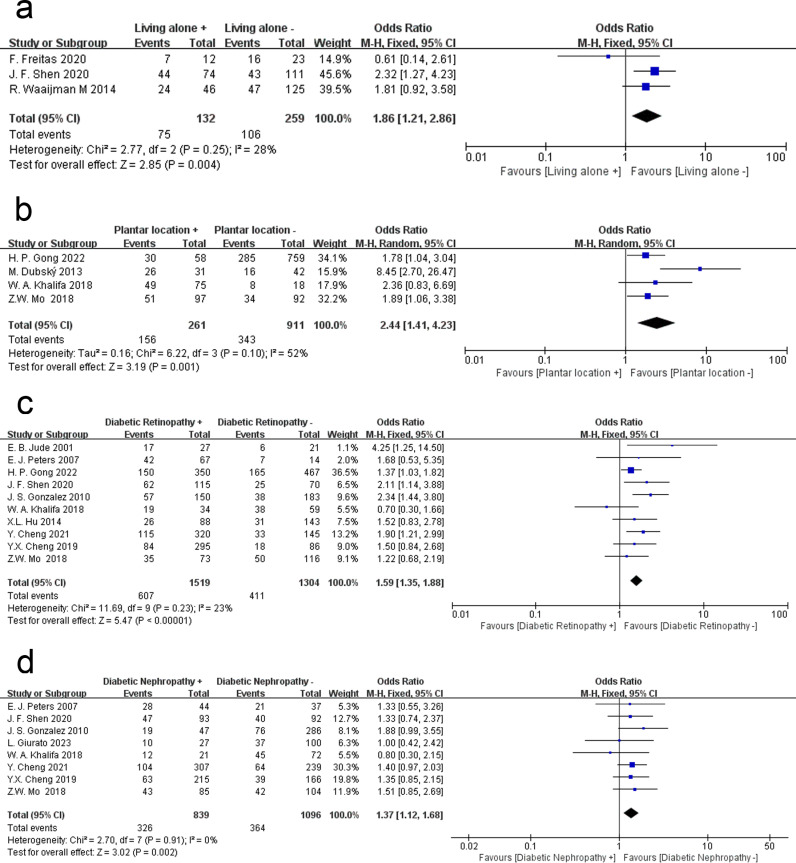
Meta-analysis results for the recurrence of DFUs between the two groups. (a) Living alone; (b) The location of the ulcer; (c) Diabetic retinopathy; (d) Diabetic nephropathy.

A fixed effects model was used to examine the relationship between living alone and recurrence among DFU patients (P = 0.25, I^2^ = 28%). Across three studies, the results showed that DFU patients who lived alone had a higher incidence of recurrence (OR = 1.86, 95% CI = 1.21 ~ 2.86, P = 0.004) (Fig 3a).

### The location of the ulcer

The location of ulcer may affect the prognosis of the ulcer and the risk of recurrence. We explored whether plantar location affects recurrence in DFU patients. Relevant data from four studies were analyzed by a random effects model (P = 0.10, I^2^ = 52%), and the results indicated that plantar ulcers had a higher risk of recurrence (OR = 2.44, 95% CI = 1.41 ~ 4.23, P = 0.001) (Fig 3b).

### Diabetic retinopathy

A fixed effects model was used to determine whether DFU recurrence was associated with diabetic retinopathy (P = 0.23, I^2^ = 23%). Data from 10 studies indicate that diabetic retinopathy is associated with an increased risk of recurrence in DFU patients (OR = 1.59, 95% CI = 1.35 ~ 1.88, P < 0.00001) (Fig 3c).

### Diabetic nephropathy

The diagnosis of diabetic nephropathy is determined by 24-hour urinary albumin excretion and serum creatinine levels. A fixed effects model was used to determine the relationship between the presence of diabetic nephropathy and recurrence among DFU patients (P = 0.91, I^2^ = 0%). Across eight articles, the incidence of ulcer recurrence was found to be associated with the presence of diabetic retinopathy in DFU patients (OR = 1.37, 95% CI = 1.12 ~ 1.68, P = 0.002) (Fig 3d).

### Diabetic peripheral neuropathy

**Fig 4:**
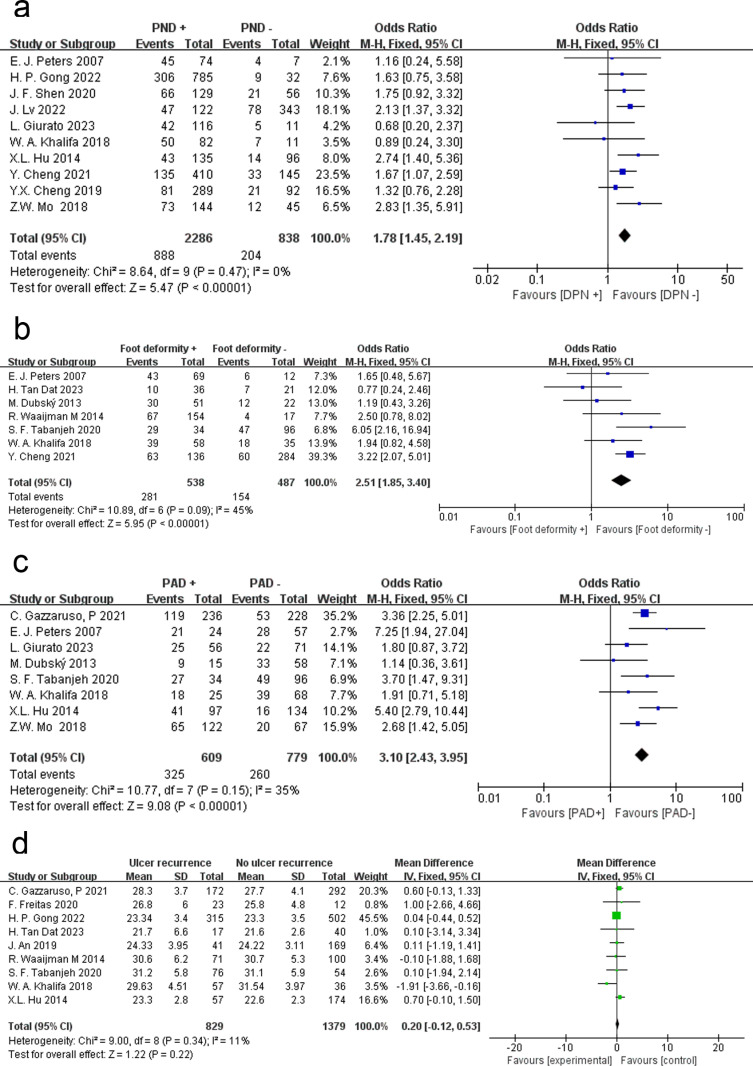
Meta-analysis results for the recurrence of DFUs between the two groups. (a) Diabetic peripheral neuropathy; (b) Foot deformity; (c) Peripheral arterial disease; (d) Body mass index.

A total of 10 papers examined the relationship between diabetic peripheral neuropathy (DPN) and DFU recurrence. A fixed effects model was used because of the low level of heterogeneity between studies (P = 0.47, I² = 0%). We found that DFU patients with peripheral neuropathy had a higher risk of ulcer recurrence (OR = 1.78, 95% CI = 1.45 ~ 2.19, P < 0.00001) (Fig 4a).

### Foot deformity

This study also analyzed whether foot deformity affects the recurrence rate of foot ulcers in DFU patients. Seven studies were analyzed by a fixed effects model (P = 0.09, I^2^ = 45%), and the results showed that foot deformities increased the risk of ulcer recurrence (OR = 2.51, 95% CI = 1.85 ~ 3.40 P < 0.00001) (Fig 4b).

### Peripheral arterial disease

Peripheral arterial disease (PAD) is one of the most common complications in patients with DFU. A fixed effects model was used to analyze the data from 8 articles (P = 0.15, I^2^ = 35%). The results showed that peripheral vascular diseases significantly increased the risk of recurrence of foot ulcers in DFU patients (OR = 3.10, 95% CI = 2.43 ~ 3.95 P < 0.00001) (Fig 4c).

### Body mass index

Nine articles examined body mass index (BMI), and the data were analyzed by using a fixed effects model (P = 0.34, I^2^ = 11%). The analysis showed that BMI was not associated with the risk of recurrence among DFU patients (MD = 0.20, 95% CI = −0.12 ~ 0.53, P = 0.22) (Fig 4d) Table 2.

**Table 2:** Pooled outcomes of all factors.

Risk factors	Studies	Sample size (RDFU/total)	Statistical model	OR/MD	95% CI	P*-*value	Risk factor *Yes/No*
Sex	19	1546/4356	Fixed-effects	1.26	1.10 ~ 1.44	0.0009	*Yes*
Hypertension	8	845/2160	Fixed-effects	1.16	0.96 ~ 1.40	0.13	*No*
Smoking history	16	1496/4318	Fixed-effects	1.18	1.04 ~ 1.35	0.01	*Yes*
Living alone	3	181/391	Fixed-effects	1.86	1.21 ~ 2.86	0.004	*Yes*
Plantar ulcer	4	499/1172	Random-effects	2.44	1.41 ~ 4.23	0.001	*Yes*
Diabetic retinopathy	10	1018/2823	Fixed-effects	1.59	1.35 ~ 1.88	<0.00001	*Yes*
Diabetic nephropathy	8	690/1935	Fixed-effects	1.40	1.13 ~ 1.72	0.002	*Yes*
Diabetic neuropathy	10	1092/3124	Fixed-effects	1.78	1.45 ~ 2.19	<0.00001	*Yes*
Foot deformity	7	435/1025	Fixed-effects	2.51	1.85 ~ 3.40	<0.00001	*Yes*
Peripheral arterial disease	8	585/1388	Fixed-effects	3.10	2.43 ~ 3.95	<0.00001	*Yes*
Body mass index	9	829/2208	Fixed-effects	0.20	−0.12 ~ 0.53	0.22	*No*

## Discussion

The recurrence of ulcers has been confirmed to be related to pathophysiological indicators of limbs, mainly including ischemia, infection, DPN and foot biomechanical load abnormalities [33]. Some studies have identified risk factors for the recurrence of DFUs. We summarized these studies and extracted relevant data. This meta-analysis identified relevant factors affecting the recurrence of DFU.

Similar meta-analyses about the recurrence of DFUs have been published, and one of them analyzed the odds ratio (OR) and 95% confidence intervals rather than extracting specific population data related to the risk factors for DFU recurrence [7]. Another meta-analysis extracted detailed data, but only 9 articles were included [34]. Therefore, previous meta-analyses included too few studies and extracted data, which lead to unavoidable significant heterogeneity. To obtain more credible conclusions and avoid the limitations of the previous meta-analyses, our study extracted detailed data and analyzed more eligible studies. We supplemented the previous research conclusions and observed some new findings.

Sex has been found to be related to the occurrence and progression of many diseases. In previous studies, we found that male patients with DFU had a higher incidence of amputation [35]. In this study, we found that male patients with DFU were also more likely to experience recurrence. A total of 2765 male patients and 1591 female patients were examined in the included studies. A total of 1034 male patients and 512 female patients experienced DFU recurrence. The results of our analysis revealed that compared to women, male DFU patients have a 1.26-fold higher risk of ulcer recurrence. The reason for this difference is currently uncertain. One explanation for this difference may be that male patients place less emphasis on foot health than female patients: men do not pay as much attention to changes in their feet as women do, and they may be more inclined to ignore foot problems for a longer period of time [36]. Previous studies have confirmed that the pain response of females is more variable and sensitive than that of males, making it easier for females to detect physical abnormalities [37,38]. For these reasons, men may discover the occurrence or recurrence of foot ulcers later than women, which may ultimately affect the outcome of DFU.

The negative effects of smoking on patients with diabetes have been widely confirmed [39,40]. Unfortunately, smoking is still a common phenomenon among DFU patients: of the 4318 patients with DFU in this meta-analysis, 1900 patients (44.00%) were still smoking or had a history of smoking. Our study found that compared to patients who have never smoked before, patients who are currently smoking or have a history of smoking have an 18% higher recurrence rate. The studies included in this meta-analysis only assessed the impact of smoking history on ulcer recurrence, which may weaken the impact of tobacco on the prognosis of ulcers. These specific smoking characteristics, such as smoking frequency, smoking volume, smoking duration, and tobacco type, may have a greater impact on the recurrence of ulcers, which requires more studies and detailed data to support the conclusion.

For people at risk of foot ulcers associated with diabetes, foot self-care can be performed at a lower cost, which is a strong recommendation in the 2023 International Working Group on the Diabetic Foot (IWGDF) guideline on the prevention of foot ulcers in persons with diabetes [41]. Qualitative and quantitative studies have found that family and friends are helpful to diabetes patients’ self-care behavior, indicating that social support is crucial to promote diabetes self-management behavior [42,43]. Patients living with their families have relatively complete family functions and high levels of social support, which may be beneficial for their self-care behavior. Although only a few diabetic foot patients in our study lived alone, the lack of social and family support had a negative impact on the prevention and healing of diabetic foot ulcers.

In our study, one of the high-risk factors associated with ulcer recurrence was the plantar position of the initial ulcer. A possible explanation for this finding is that ulcers located on the plantar of the insensitive foot are exposed to repeated injuries and subjected to higher pressure than ulcers in other parts [44]. Based on the impact of plantar pressure on the occurrence and recurrence of ulcers, some studies suggest that patients will benefit from customized shoes that can improve plantar pressure [20,45]. Foot deformity is another high-risk factor associated with ulcer recurrence, which can alter the distribution of foot pressure and weaken the fit between the foot and the shoes. A previous meta-analysis found that the relationship between foot deformity and DFU recurrence was not statistically significant, but this conclusion was only based on two studies [7]. Our study included more studies and analyzed more detailed data. DFU patients with foot deformity had a higher risk of recurrence than DFU patients without concomitant deformities, with an OR value of 2.51.

Diabetic retinopathy, diabetic nephropathy and diabetic peripheral neuropathy are considered complications of diabetic microvascular disease. A considerable number of DFU patients suffer from diabetic microvascular disease. The benefits of glycemic control are obvious in diabetes microvascular disease but not in macrovascular outcomes [46], which means that patients with diabetes microvascular disease often have a worse glycemic control. Glycemic control plays an important role in the management of diabetic foot and affects the healing and prognosis of DFU [47,48]. Diabetic peripheral neuropathy is believed to play an important role in the occurrence and recurrence of foot ulcers. Patients with diabetic neuropathy lack warning symptoms related to pain and may not take appropriate preventive measures, such as wearing appropriate shoes as needed [20]. Recurrent injuries, especially those related to foot pressure points, may also be difficult to detect, thereby promoting the occurrence of ulcers. In addition, autonomic neuropathy leads to microvascular dysfunction. Impaired nociceptive reflex and reduced inflammatory response [49]. Complications related to sensory, autonomic and motor neuropathy are involved in the pathogenesis of typical DFU, thus leading to ulcer recurrence due to the irreversible process of the disease [3].

The presence of PAD is an important consideration in the management of diabetic foot ulcers. Lower limb ischemia and malnutrition caused by PAD are important reasons for the occurrence and recurrence of diabetic foot-related ulcers, and they are also factors in the pathogenesis of typical diabetic foot ulcers [3]. DFU patients with and without PAD differ in clinical characteristics, outcomes and predictors of outcome [50]. The ischemia caused by PAD inhibits wound healing. Wound healing may be further impaired by the development of infection and gangrene. Therefore, diabetic patients may have ulcers. Although active local treatment measures have been taken, the ulcers still cannot heal. In addition, the patient may have progressive gangrenous changes in the foot, resulting in a limb-threatening condition [51]. Unfortunately, PAD is very common in patients with diabetic foot. The macrovascular complications of diabetes are considered to be caused by the adverse effects of hyperglycemia, with a 28% risk increase for every 1% increase in glycosylated hemoglobin (HbA1c) [52]. For diabetic individuals, it is advised that their HbA1c level be kept below 8.0% in order to avoid complications and death [53]. Revascularization in patients with a TcPO2 < 30 mmHg, including intravascular intervention and open surgery, can restore blood flow and improve the healing ability of DFU compared to those patients without undergoing revascularization [54,55]. Therefore, DFU patients with peripheral artery disease can benefit from using the WIfI (wound, ischemia, foot infection) system to stratify the healing possibility and amputation risk [56].

There are many factors that affect the ulcer recurrence of diabetes foot patients after ulcer healing, and our research shows only a part of them. These risk factors are inherent to patients, many of which are immutable, such as gender, smoking history, and diabetes complications that have occurred. At present, the focus of research is how to promote the healing of foot ulcers in diabetes. Although there is still limited research on finding interventions to reduce ulcer recurrence, some interventions have been proven to potentially benefit patients with healed DFUs. Pressure-relieving shoes or orthotics that accommodate the foot shape and any deformities can effectively reduce the risk of ulcer recurrence, including customized shoes or insoles for ultra deep shoes [41,57]. People who combine those risk factors and healed foot ulcers may benefit from strict screening and professional foot care every 1 to 3 months [41]. A comprehensive therapeutic patient education was reported to significantly reduce DFUs recurrence [58]. therefore, professional supervision and education should be considered for patients with risk factors. Dermal thermometry is useful in determining the area of pre-ulcer inflammation. For patients with healed ulcer, skin temperature monitoring can advance intervention and reduce the risk of ulcer recurrence [59] In addition, compared with routine care, monitoring skin temperature at home to prevent DFUs is at best equally cost-effective [60]. Other interventions, such as nerve decompression surgery and reconstructive foot/ankle surgery, may also benefit patients who meet surgical requirements [61,62].

## Limitations

This study has several limitations that must be considered when interpreting the results. There is no accepted method to determine the quality of research methods or the risk of bias. Although the Cochrane Group advocates the use of the NOS, different quality assessment tools may produce different results. There were 22 papers included in our meta-analysis, but most of them were retrospective studies. Half of the included studies were from China, which may have caused bias. Different hospitals in different regions have different diagnostic and therapeutic capabilities for patients, which may affect the prognosis of patients. Several included studies contained significantly more patients than others, and these trials may lead to bias in assessing the outcome of our study. Although some studies have reported other factors affecting the recurrence of foot ulcers in diabetes, due to too few data and enormous heterogeneity, we failed to analyze these possible risk factors, including history of amputation, cardiovascular history, history of vascular intervention, duration of diabetes, HbA1c, and white blood cell count. The combination of multiple risk factors may increase the chance of ulcer recurrence, but due to the lack of relevant research, we did not conduct targeted analysis in this study. Due to the existence of recent relevant meta-analysis or a lack of sufficient literature, we did not analyze the impact of post healing interventions on ulcer recurrence in our study. The differences in the study population and aims of the included studies might lead to selection bias. Diabetic foot is a long-term pathological process, and the difference in follow-up time of included studies may affect the research results.

## Conclusion

In summary, DFU has a significant impact on patients’ lives and prognosis, and recurrence can once again put patients at risk. Our meta-analysis identified the following important risk factors for DFU recurrence: male sex, smoking history, living alone, plantar ulcer, diabetic retinopathy, diabetic nephropathy, diabetic peripheral neuropathy, foot deformity, and peripheral arterial disease. Understanding these factors and their impact on ulcer recurrence is crucial for multidisciplinary teams to develop management and treatment plans for DFU patients. To prevent patients from suffering from ulcer recurrence, it is necessary to alter factors that can be reversed and strengthen supervision of factors that cannot be changed. For risk factors that cannot be altered, more frequent monitoring, foot care education, professional footwear, and early or professional guidance may also benefit patients.

## Supporting information

S1 Table PRISMA checklist. (DOC)

S2 Table Raw data 1. (XLSX)

S3 Table Raw data 2. (XLSX)

S4 Fig Raw data 3. (JPG)

S5 Fig Funnel plots. (TIF)

S6 Table Detailed Table of literature screening. (XLSX)
