# Supplementary figures and images for: Risk factors associated with the recurrence of diabetic foot ulcers: A meta-analysis
Source: PLoS One. 2025 Feb 14;20(2):e0318216. doi: 10.1371/journal.pone.0318216 (PMC11828396; doi:10.1371/journal.pone.0318216)

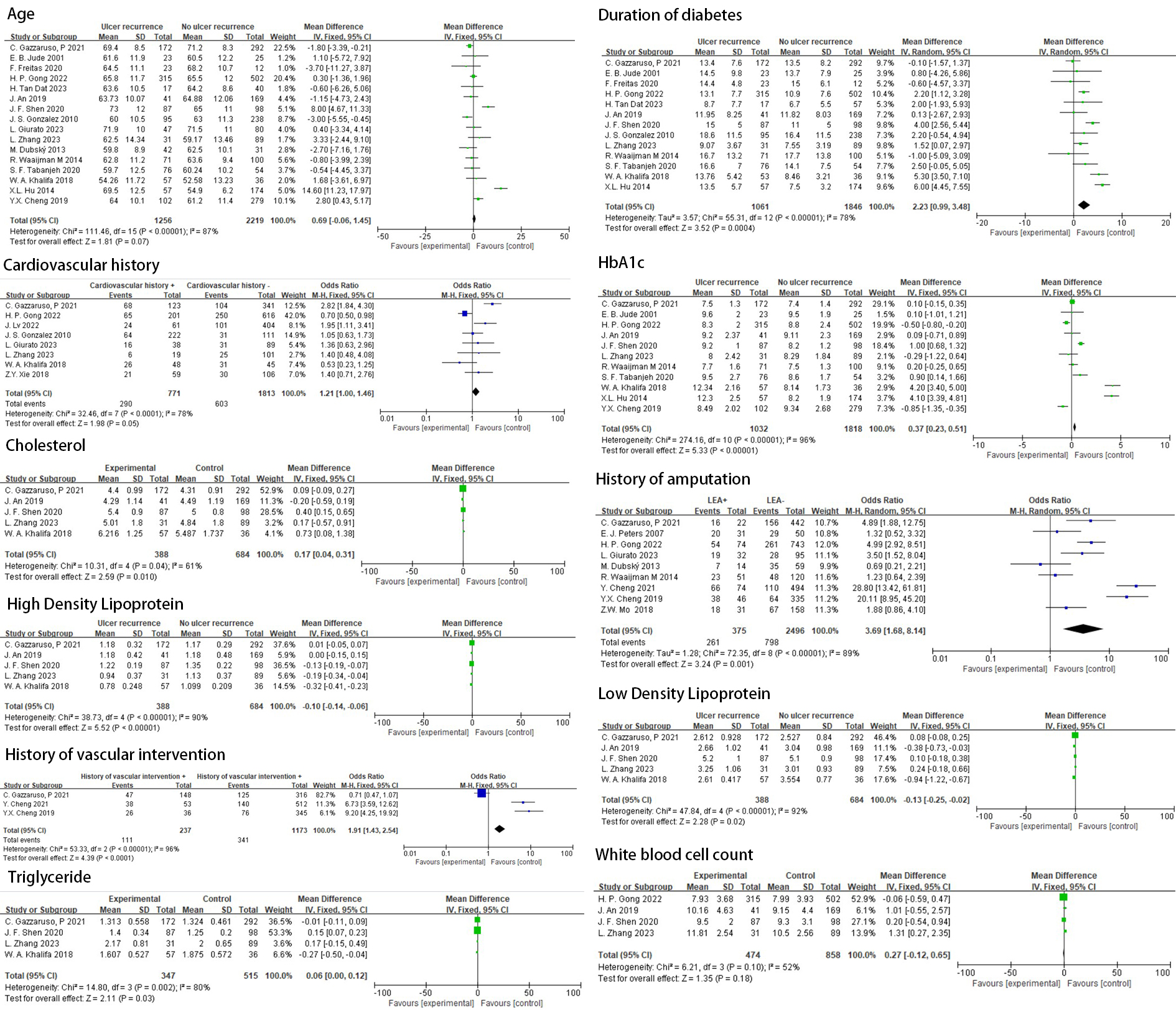

Supplement: S4 Fig — (JPG) [file pone.0318216.s004.jpg]

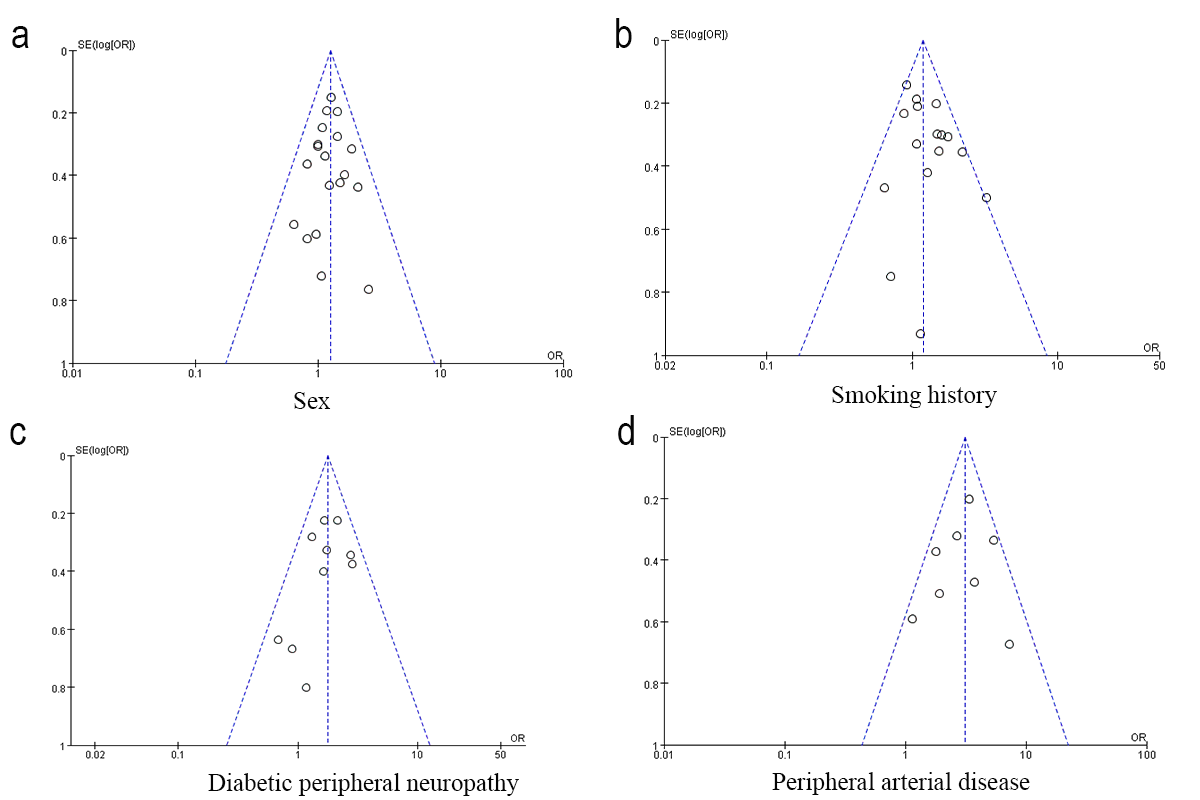

Supplement: S5 Fig — (TIF) [file pone.0318216.s005.tif]
